# An autopsy case of disseminated *Cunninghamella bertholletiae* infection in an immunocompetent patient: a case report

**DOI:** 10.1186/s12890-023-02382-y

**Published:** 2023-03-17

**Authors:** Masanori Harada, Kazuyo Yasuda, Kazumi Uruchida, Ryoma Yamashita, Keisuke Morikawa, Yutaro Ito, Eisuke Mochizuki, Shun Matsuura, Masaru Tsukui, Naoki Koshimizu

**Affiliations:** 1 Department of Respiratory Medicine, Fujieda Municipal General Hospital, Fujieda, Japan; 2 Department of Respiratory Medicine, Iwata City Hospital, Okubo, Iwata, Shizuoka, 512-3426-8677 Japan; 3 Department of Pathology, Fujieda Municipal General Hospital, Fujieda, Japan; 4 Department of Bacterial Laboratory, Fujieda Municipal General Hospital, Fujieda, Japan

**Keywords:** *Cunninghamella bertholletiae*, Immunocompetent, Colonization, Vascular invasion

## Abstract

**Background:**

Recently, deaths due to mucormycosis in immunocompromised hosts have increased; however, the clinical and pathological features of mucormycosis are not fully understood, especially in view of the associated high mortality and rare incidence in immunocompetent patients.

**Case presentation:**

We have described a rare autopsy case of a 67-year-old Japanese man with chronic obstructive pulmonary disease who contracted mucormycosis. He had not been on any immunosuppressants, and his immune functions were intact. Since 3 days prior to admission to our hospital, he had experienced progressive dyspnea, productive cough, and fever. Chest computed tomography revealed pleural effusion in the left lower hemithorax and consolidation in the right lung field. Although he was administered with tazobactam-piperacillin hydrate (13.5 g/day), renal dysfunction occurred on the ninth disease day. Therefore, it was switched to cefepime (2 g/day). However, his general condition and lung-field abnormality worsened gradually. Cytological analysis of the sputum sample at admission mainly revealed sporangiophores and unicellular sporangioles, while repeated sputum culture yielded *Cunninghamella species.* Therefore, he was diagnosed with pulmonary mucormycosis. Liposomal amphotericin B (5 mg/kg/day) was initiated on the 28^th^ disease day. However, chest radiography and electrocardiography detected cardiomegaly and atrial fibrillation, respectively, and he died on the 37^th^ disease day. A postmortem examination revealed clusters of fungal hyphae within the arteries of the right pulmonary cavity wall, the subpericardial artery, intramyocardial capillary blood vessels, and the esophageal subserosa vein. Direct sequencing revealed that all fungal culture samples were positive for *Cunninghamella bertholletiae*.

**Conclusions:**

*Cunninghamella bertholletiae* could rapidly progress from colonizing the bronchi to infecting the surrounding organs via vascular invasion even in immunocompetent patients.

## Background

Mucormycosis is an important infection that is associated with high mortality [1]. In recent decades, its incidence has increased in populations with underlying conditions, such as those with malignancies and recipients of bone marrow transplant; among these, pulmonary mucormycosis is the primary cause at initial diagnosis [2]. Among zygomycetes, *Cunninghamella spp.* are rarely isolated from samples from immunocompromised patients; however, the associated mortality is significantly higher than that associated with other zygomycetes [3]. Only few cases of disseminated pulmonary [4, 5], cardiovascular [6], and aortic [7] infections have been reported in immunocompromised patients. Furthermore, in immunocompetent patients, the occurrence of a disseminated *Cunninghamella* infection is rarer. Therefore, its clinical and pathological features are not fully understood. We experienced a case wherein an immunocompetent patient was diagnosed with disseminated *Cunninghamella bertholletiae* infection; sputum culture indicated bronchial colonization prior to the diagnosis. Histological findings from their autopsy could reveal the expected invasion sites.

## Case presentation

**Fig. 1:**
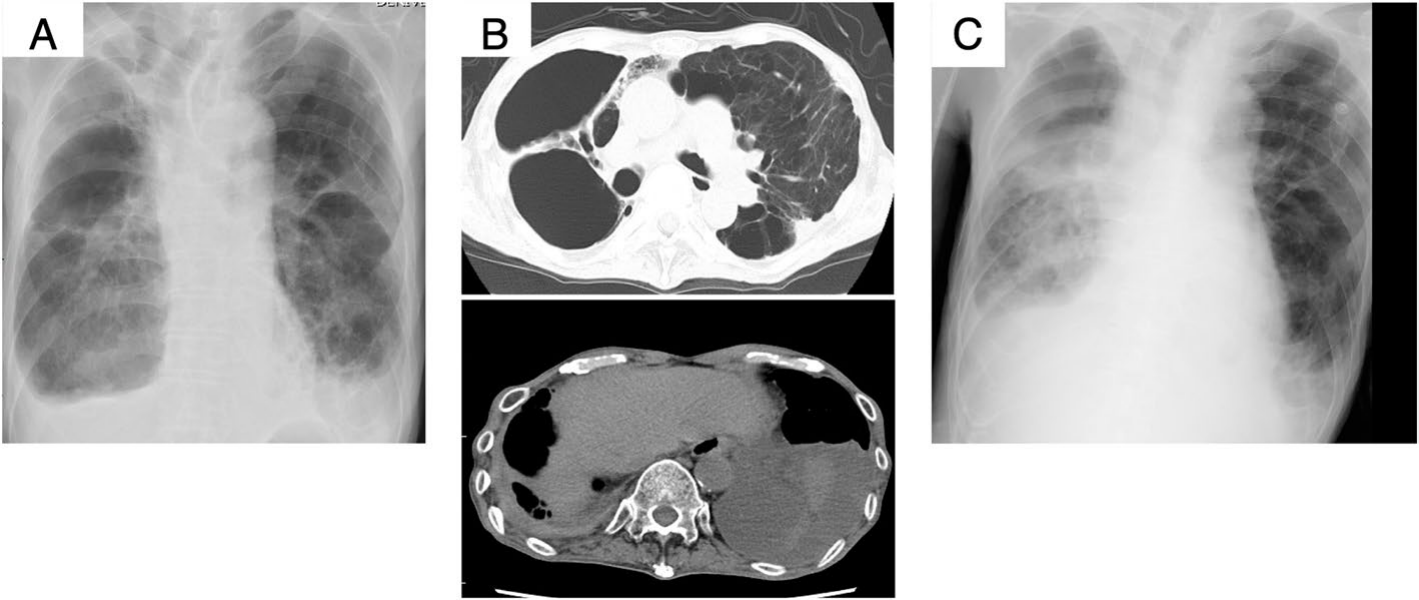
Chest radiography and computed tomography findings. Chest radiography (**a**) and chest computed tomography (**b**) findings obtained at hospitalization. Chest radiography findings obtained on the 37 ^th^ disease day (**c**)

**Fig. 2:**
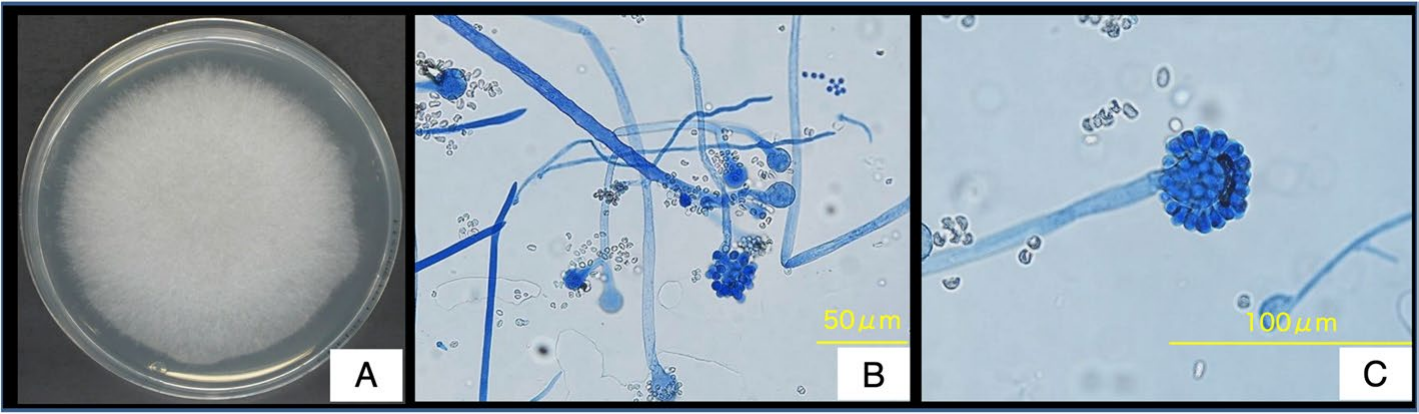
Fungus culture on potato dextrose agar (**a**). Slide culture (lactophenol cotton blue staining): low-power field (**b**) and high-power field (**c**)

**Fig. 3:**
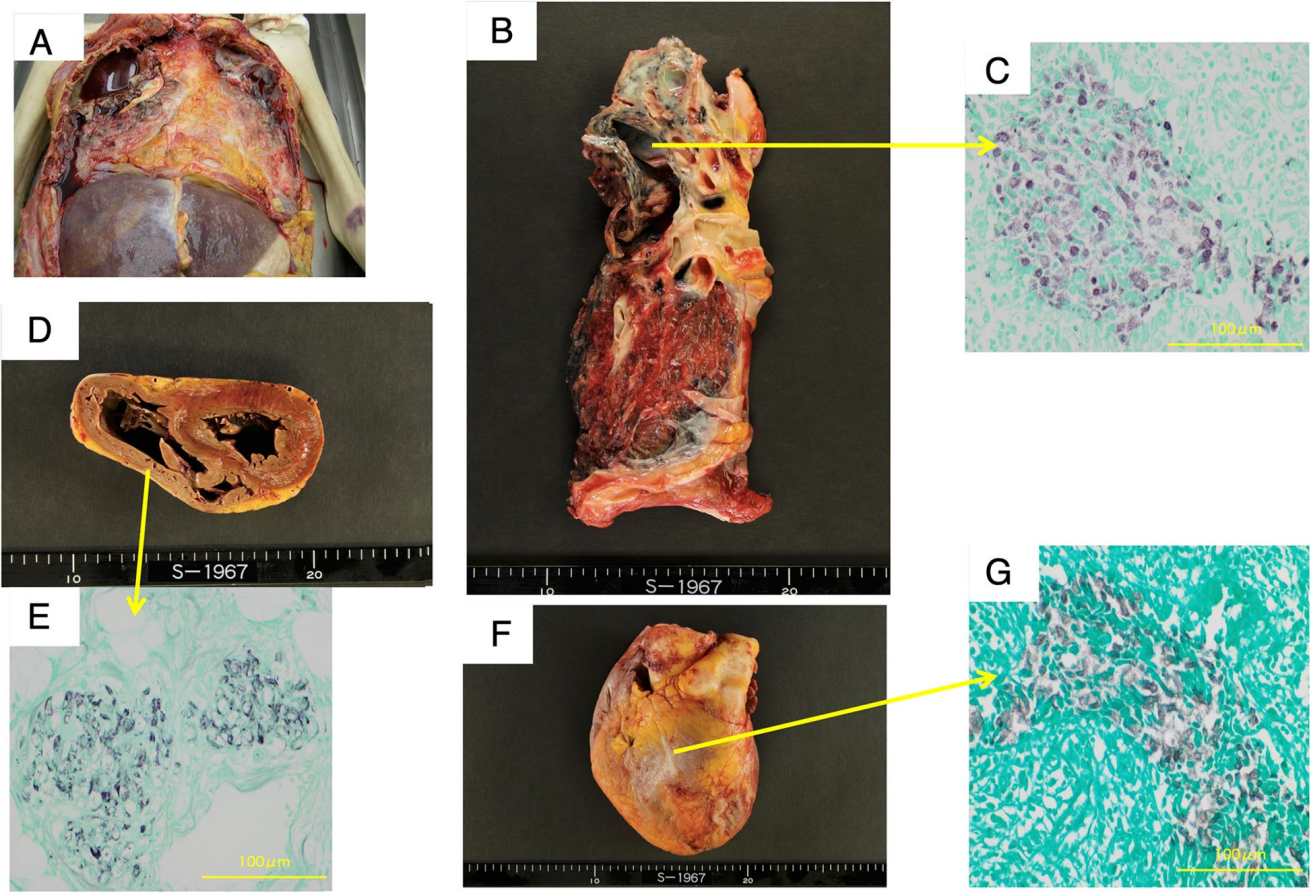
Autopsy findings: macroscopic findings of the precordium (**a**), right lung (**b**), and heart (**d** and **f**). Grocott’s staining of the intra-arterial region in the right upper lung cavity (**c**), intramyocardial capillary blood vessels (**e**), and subendocardium (**g**)

A 67-year-old Japanese man with emphysema visited our hospital every month for bronchodilator medication. He was hospitalized for progressive dyspnea, productive cough, and moderate fever that had developed 3 days prior to admission. He had a smoking habit (33 smoking pack years) and recurrent pneumothoraxes with chest tube drainage management, and a home oxygen therapy (2 L/min). He did not have a history of allergy or immunodeficiency, and did not consume alcohol as a habit. Physical examination revealed an elevated body temperature (37.8℃) and respiratory failure with SpO_2_ 97% and 3.5 L/min oxygen therapy; he also had bilateral coarse crackles without leg edema. Chest radiography revealed a bilateral consolidation shadow, with emphysema in the right lower lobe and pleural effusion in the left lung (Fig. 1A). Chest computed tomography indicated consolidation in multiple lung lobes, with left-sided pleural effusion and an adhesive collapsed lung appearance in the right upper-lung field (Fig. 1B). Temporal manual drainage was performed to relieve the left pleural exudative effusion; the sputum, blood, and pleural effusion all tested negative for a bacterial infection. Although sputum culture had yielded *Cunninghamella spp.* 6 months prior to the most recent presentation, the infection seemed to have been a respiratory tract colonization because of his good condition. Laboratory findings revealed that the only abnormalities were anemia (hemoglobin: 10.5 g/100 mL), a low albumin level (3.0 g/100 mL), and an elevated C-reaction protein level (14.0 mg/100 mL). Tests for β-d glucan and galactomannan were normal; thus, an *Aspergillus* infection was ruled out. Although there were no bacterial evidence, tazobactam-piperacillin hydrate (13.5 g/day) was administered empirically; however, it was discontinued on the ninth disease day because renal dysfunction occurred as an adverse event. The bilateral lung consolidation around the emphysema worsened gradually, and repeated sputum cultures yielded fungal agents on the 17^th^ disease day (Fig. 2A). Mass spectrometry and polymerase chain reaction (PCR)-based direct sequencing [8] revealed the pathogen to be *Cunninghamella bertholletiae* (according to the DDBJ/EMBL/GenBank [http://blast.ncbi.nlm.nih.gov/Blast.cgi] and MycoBank Database [http://www.mycobank.org/]). Cytological analysis mainly revealed sporangiophores, with all branches swelling up to vesicles producing unicellular sporangioles (Fig. 2B, C). These findings were consistent with a pulmonary *Cunninghamella* infection. Although liposomal amphotericin B (5 mg/kg/day) was administered since the 28^th^ disease day in addition to cefepime (2 g/day), chest radiography and electrocardiography revealed cardiomegaly and atrial fibrillation on the 29^th^ disease day, respectively. The serum brain natriuretic peptide (BNP) level was also elevated (956 pg/mL). Respiratory dysfunction occurred gradually and the plasma BNP level increased to 2,338 pg/mL. The patient died from multiorgan dysfunction on the 37^th^ disease day (Fig. 1C). Macroscopically, a postmortem examination (Fig. 3A) revealed a cavity region and coagulative necrosis in the right upper lung, a small amount (50 mL) of bilateral pleural effusion, and pericardial fluid (50 mL). Histopathological examination revealed a cluster of fungal hyphae within the arteries of the right cavity wall (Fig. 3B), subpericardial artery (Fig. 3C), intramyocardial capillary blood vessels (Fig. 3D–G), and esophageal subserosa vein. The fungus cultures from the right upper bronchial secretion, pleural, and pericardial fluid were positive for *Cunninghamella bertholletiae* (Fig. 3A, B, F). These findings suggested that the colonized *Cunninghamella bertholletiae* in the upper bronchus had invaded the blood vessels and disseminated to other organs, and myocardial invasion had resulted in the critical damage that led to the death in this case.

## Discussion and conclusions

This was a rare case of pulmonary *Cunninghamella bertholletiae* infection that occurred in an immunocompetent patient. Most *Cunninghamella* infections tend to occur in immunocompromised hosts, including transplantation recipients and patients with hematological malignancies [9]. Recently, *Cunninghamella* was reported to have accounted for only 7% of all mucormycosis cases and isolated primarily from patients with pulmonary or disseminated disease. The associated mortality was significantly higher than that associated with other Mucorales (71% [23/30] vs. 44% [185/417]; *p* < 0.001) [1]. However, the pathophysiology has not been fully understood because of its rarity and rapid disease progression in immunocompromised patients.

Only two cases of pulmonary *Cunninghamella bertholletiae* infection have been reported in immunocompetent patients [10, 11]. One of these occurred in a 61-year-old White man with a history of alcoholic binges who had right pleural effusion, pneumothorax, and a right upper lobe cavitary lesion. *Cunninghamella bertholletiae* was detected in the abscess wall and surrounding lung parenchyma sections without vascular involvement or dissemination. The lung deformity may have promoted the growth of *Cunninghamella bertholletiae*, similar to in our case [10]. The other case was of a 74-year-old man with exacerbated asthma–chronic obstructive pulmonary disease overlap syndrome, who was diagnosed with allergic bronchopulmonary mycosis; tests detected a prolonged serum-specific immunoglobulin type E antibody against mucormycetes. This indicates the possibility of *Cunninghamella bertholletiae* having contaminated his organs (such as the respiratory tract) or emphysema [11]. Both cases suggest the colonization of *Cunninghamella bertholletiae* with pulmonary deformation, as in our case, which is sometimes misdiagnosed as a contamination.

Distinguishing between a definite diagnosis of mucormycetes and colonization seems rather difficult. Therefore, the detailed morphological features were investigated in this case. Generally, temperature plays an important role in fungal growth; the optimal growth temperature for *Cunninghamella elegans* is known to be approximately 35℃, which is lower than the human body temperature or the temperature of the respiratory tract. Conversely, for other *Cunninghamella spp.* (such as *Cunninghamella bertholletiae* and *Cunninghamella echinulata*), the best growth temperature is higher than that for *Cunninghamella elegans*, and these species are thought to have thermotolerance [12]. In our case, biological culture examination suggested that the *Cunninghamella bertholletiae* infection occurred prior to admission to our hospital; the pathogen may have colonized the respiratory tract for a long time and grown gradually to invade the cardiovascular system until the critical stage of death. In immunocompetent patients in particular, *Cunninghamella bertholletiae* might likely colonize the respiratory tract.

To detect mucormycetes at the earliest stage, a novel DNA sequencing method has been suggested [13]. In this present case, the serum *Cunninghamella bertholletiae* DNA load was measured using quantitative PCR. The copy number on the day of the patient’s death was higher than that on the onset day. This suggests that quantification of *Cunninghamella bertholletiae* DNA in the serum could be useful for the diagnosis and evaluation of mucormycosis. Because the DNA is not detected in healthy or non-pathological patients, this would be a useful way for distinguishing between definite infection and colonization.

In our case, we observed biological and pathological evidence in autopsy tissues from several organs in an immunocompetent patient. This finding is indicative of the common invasion sites of *Cunninghamella bertholletiae* itself. Fungal hyphae were found within the pulmonary cavity wall, subpericardial artery, intramyocardial capillary blood vessels, and esophageal subserosa veins (Fig. 3); this is in line with previous reports [6, 7, 11, 14]. Pulmonary and cardiovascular deformities are considered common invasive regions [11]; cardiovascular invasion sometimes follows a critical course in the presence of a *Cunninghamella* infection, with arrhythmia or acute exacerbation of heart failure, as in our case. Only two cases of cardiovascular invasion with a large mass within the left inferior wall of the ventricular cavity have been reported; one involved hypokinesis [6] and the other involved invasion of the septate hyphae of *Cunninghamella spp.* into the vascular wall [7]. Both patients in these cases died, and an autopsy was performed.

In conclusion, we have presented a rare case of an *Cunninghamella bertholletiae* infection that occurred in an immunocompetent patient and followed a critical course even under antifungal treatment. Because useful diagnostic markers are lacking, it is difficult to distinguish between colonization and definite diagnosis in order to initiate antifungal treatment at an earlier stage. This pathogen can rapidly progress from colonizing the bronchi to infecting the surrounding organs via vascular invasion even in immunocompetent patients.

## Data Availability

All data supporting our findings are included in this published article.

## Abbreviation

PCR: Polymerase chain reaction
